# The more we learn, the more diverse it gets: structures, functions and evolution in the Phosphofructokinase Superfamily

**DOI:** 10.1042/BCJ20253024

**Published:** 2025-05-06

**Authors:** Jordan A. Compton, Wayne M. Patrick

**Affiliations:** 1 School of Biological Sciences, Victoria University of Wellington, PO Box 600, Wellington 6140, New Zealand

**Keywords:** allosteric regulation, enzyme evolution, glycolysis, kinetics, phosphate donor specificity, structural biology, substrate specificity

## Abstract

The enzyme 6-phosphofructokinase (PFK) phosphorylates d-fructose 6-phosphate, producing d-fructose 1,6-bisphosphate. The canonical version—discovered almost 90 years ago—is ATP-dependent, allosterically regulated and catalyses the first committed step in glycolysis. However, beyond this textbook enzyme, there is fascinating functional and structural variety among PFKs across the tree of life. While PFKs are found in two non-homologous superfamilies, here, we review the universally distributed enzymes in one, the Phosphofructokinase Superfamily. We focus on summarising the diversity within this superfamily. A key partition regards the identity of the phosphate donor, which can be ATP or inorganic pyrophosphate (PP_i_). Considerable insights into functional and evolutionary aspects of the ATP- and PP_i_-dependent PFKs have come through structural biology, with 45 structures now available in the Protein Data Bank. One recent highlight was the use of cryoEM and molecular dynamics simulations to illuminate the structural basis of allosteric regulation in human liver PFK. Others were to explore interactions of drug-like small molecules with the PFKs from *Trypanosoma brucei* and human liver, revealing new routes to antibiotics and immune modulators, respectively. In contrast with the ATP-dependent enzymes, PP_i_-dependent PFKs are typically non-allosteric and catalyse a readily reversible reaction. Some also play an additional physiological role by phosphorylating d-sedoheptulose 7-phosphate. We discuss why these properties are plausibly ancestral. Finally, we also emphasise how much remains to be discovered. For example, the 45 experimentally determined structures are from only 14 species. Nine decades in, it is still a great time to be studying PFK.

## Introduction: a textbook structure–function relationship

The enzyme 6-phosphofructokinase (PFK) was discovered almost 90 years ago in the laboratory overseen by Jakub Karol Parnas [1]. d-Fructose 1,6-bisphosphate (FBP)—the Harden–Young ester—had already been isolated and, in fact, it was the first metabolic intermediate ever to be identified [2]. Thus, it was a major milestone in elucidating the Embden–Meyerhof–Parnas pathway of glycolysis when PFK was revealed as the enzyme responsible for forming FBP, from d-fructose 6-phosphate (F6P) [3].

The earliest characterised versions of PFK use ATP to phosphorylate F6P and are members of Enzyme Commission (EC) class 2.7.1.11. These ATP-dependent PFKs also play important regulatory roles in controlling flux through glycolysis. They catalyse the first committed step in the pathway, thereby committing the cell to use glucose for producing energy. As originally discussed in exquisite detail by Monod and his co-workers, who were studying the enzyme from *Escherichia coli*, PFKs are textbook examples of co-operative kinetics (with respect to F6P concentration) and allosteric regulation [4]. ADP and GDP are powerful allosteric activators of *E. coli* PFK (*Eco*PFK), while phosphoenolpyruvate is an allosteric inhibitor [4]. This allosteric regulation tunes glycolytic flux to reflect the cell’s energy and metabolic requirements.

**Figure 1:**
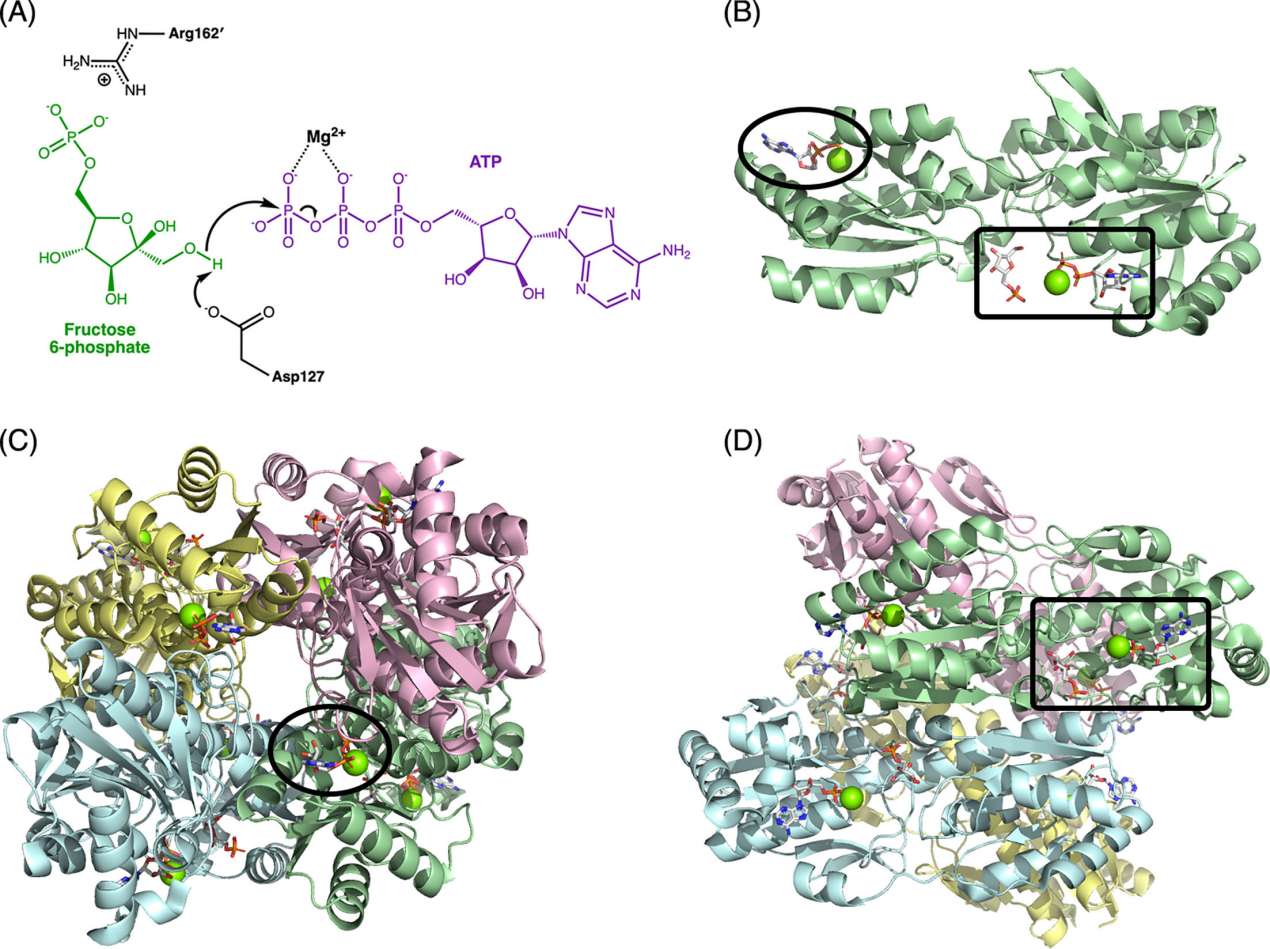
Structure and function in the bacterial ATP-dependent PFKs. (**A**) Simplified mechanism for PFK-catalysed phosphoryl transfer. The general base catalyst is Asp127 in both the *E. coli* and *G. stearothermophilus* PFKs (*Eco*PFK and *Gst*PFK, respectively). Arg162´ from a neighbouring subunit, which interacts with F6P in the active R state but not the T state, is also shown. In the R state *Gst*PFK structure (PDB ID: 4PFK; [6]), the two N–O distances between Arg162´ and the nearest phosphate oxygens on F6P are ~3 Å. (**B**) Two-domain structure of one protomer from *Gst*PFK (PDB ID: 4PFK), with F6P, ADP and Mg^2+^ in the active site (rectangle), and ADP and Mg^2+^ in the effector site (oval). (**C**) The *Gst*PFK tetramer (PDB ID: 4PFK), with each chain coloured differently. The oval highlights the location of the same effector site from (**B**), now revealed to be at the interface of the subunits coloured green and pink. (**D**) A second orientation of the tetramer from panel (**C**), emphasising the location of the active site from (**B**) at a different interface (green- and blue-coloured subunits) from the effector site in (**C**). F6P, fructose 6-phosphate; PFK, 6-phosphofructokinase.

Given PFK’s role at the critical control point in glycolysis, and as a paradigm of allostery, it is not surprising that it was also one of the first enzymes to have its structure determined by X-ray crystallography and then to be probed by site-directed mutagenesis. In a pioneering series of papers, Evans and his group determined structures of the homotetrameric PFKs from the bacteria *Geobacillus stearothermophilus* and *E. coli* [5-7]. Each subunit in the tetramer comprises two Rossmann fold domains. The larger domain binds the substrate ATP, while the smaller one binds F6P (Figure 1B). The F6P-binding domain also contains the binding site for allosteric effectors, at the C-terminal helix (Figure 1B). In the tetramer, each subunit mainly contacts two others, with one contact contributing to the effector site, and the other contributing to the active site (Figure 1C and D). The structures revealed the key active site residues, which were subsequently probed by site-directed mutagenesis of *Eco*PFK [8,9]. These mutagenesis studies confirmed a catalytic mechanism for phosphoryl transfer involving direct nucleophilic attack of the F6P 1-hydroxyl on the γ-phosphate of ATP. A general base catalyst (Asp127 in *Eco*PFK) increases the nucleophilicity of the F6P hydroxyl group by abstracting its proton (Figure 1A).

Iconically, Evans et al. determined *G. stearothermophilus* PFK (*Gst*PFK) structures in both the active, high-affinity R state and the allosterically inhibited T state. The R-to-T transition is associated with a 7° rigid body rotation of one dimer of subunits, with respect to the other dimer. This alters interactions at subunit interfaces, which are communicated to the active sites in the tetramer [10]. In the R state, Arg162´ (with the prime indicating an amino acid from a neighbouring subunit) is one of four residues that bind the phosphate of F6P, via a salt bridge (Figure 1A). However, Arg162´ is located at the end of a helical turn that unwinds upon transition to the T state. This causes Arg162´ to swing away so that it is replaced in the active site by Glu161´. The arrival of a negatively charged side chain in the phosphate binding site causes reduced affinity for F6P in the T state. This unwinding is also affected by the binding of molecules in the effector site: unwinding is prevented by the binding of ADP [10].

However, there is significantly more diversity among the structures, functions and regulatory mechanisms of PFK enzymes than these classic studies (and most textbooks) suggest. The purpose of this review is first to differentiate between the two non-homologous superfamilies of PFKs and then to summarise the diversity within the predominant superfamily. While we discuss the diverse sequences, functions and evolutionary events within the superfamily, a primary focus is on insights that are emerging from the increasing number of experimentally determined PFK structures.

### PFKs are found in two non-homologous superfamilies

**Figure 2:**
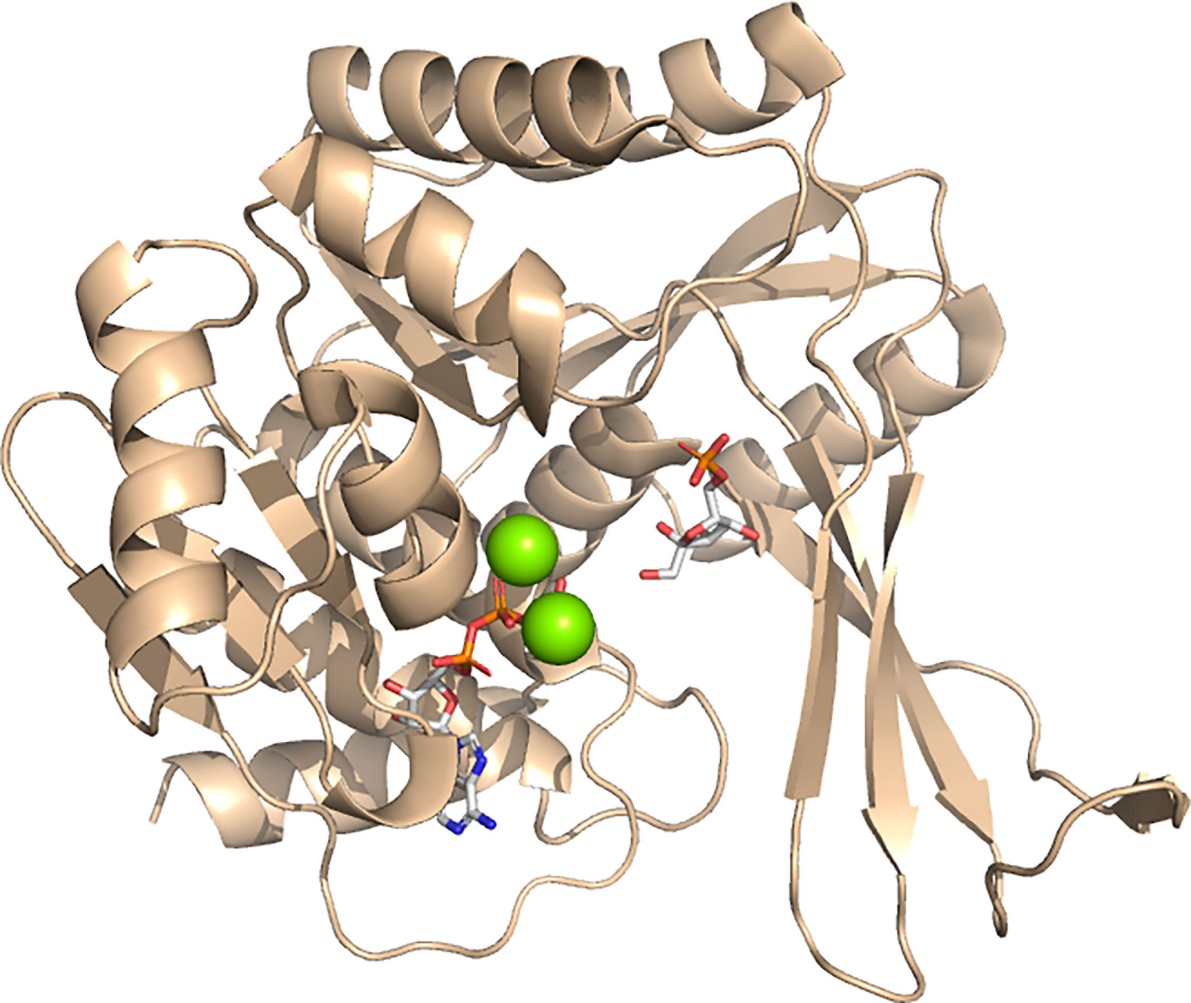
A PFK from the Ribokinase-Like Superfamily. Chain A of the homodimeric *E. coli* PFK-2 (PDB ID: 3UQD; [14]) is shown. The active site contains ATP and F6P (white sticks) and two Mg^2+^ ions (green spheres) and is located in a Rossmann-like, three-layered α/β/α sandwich domain that characterises the Ribokinase-Like Superfamily. Comparison with Figure 1B reveals the overall lack of similarity with the two-Rossmann structures of the Phosphofructokinase Superfamily. F6P, fructose 6-phosphate; PFK, 6-phosphofructokinase.

The focus of this review is on the broadly distributed, Rossmann fold-containing superfamily of PFKs. In the InterPro database [11], this group of homologous enzymes is named the Phosphofructokinase Superfamily and it is database entry IPR035966. This superfamily is sometimes referred to as the PFKA family [12,13]. However, it is also important to note that there is a second class of PFKs, sometimes named the PFKB family [12,13]. The PFKB enzymes are not homologues of superfamily IPR035966 (Figure 2). Instead, they are members of the Ribokinase-Like Superfamily of sugar kinases [15], which has InterPro entry number IPR029056. This superfamily contains the minor ATP-dependent PFK from *E. coli* (PFK-2), as well as the ADP-dependent PFKs from archaea (EC 2.7.1.146) [16]. The Ribokinase-Like Superfamily also contains bacterial and eukaryotic 1-phosphofructokinases (enzymes that phosphorylate fructose 1-phosphate to form FBP), ribokinases, phosphotagatokinases, adenosine kinases and others [15,17]. The interested reader is referred elsewhere for more detailed discussions of the Ribokinase-Like Superfamily [16-18].

### The murky world of the Phosphofructokinase Superfamily

Phylogenetic analyses continually confirm a common evolutionary ancestor for the members of the Phosphofructokinase Superfamily [12,19-25]. At the same time, complex patterns of evolution have yielded substantial diversity in the specificity, regulation and structural features of superfamily members. In general, classifying and organising the superfamily has therefore proven complicated.

A significant partition in the superfamily arises from the identity of the phosphate donor. All PFKs were assumed to utilise ATP until 1974, when an enzyme from the amoeba, *Entamoeba histolytica*, was found to use inorganic pyrophosphate (PP_i_) instead [26]. More PP_i_-dependent PFKs have since been discovered in plants, anaerobic bacteria, archaea and unicellular eukaryotes. These enzymes belong to EC class 2.7.1.90, rather than EC 2.7.1.11 like the ATP-dependent PFKs. While the EC numbers are useful descriptors, one caveat is that they are based on function and do not imply homology. Thus ATP-dependent PFKs from the Ribokinase-Like Superfamily, such as *E. coli* PFK-2, are also members of EC class 2.7.1.11. At the time of writing (November 2024), the BRaunschweig ENzyme DAtabase (BRENDA) lists 381 functional parameters for the PP_i_-dependent PFKs in EC 2.7.1.90, compared with 717 for the ATP-dependent enzymes in EC 2.7.1.11 [27].

Many ATP-dependent eukaryotic PFKs are significantly more complex than their bacterial homologues such as *Gst*PFK (Figure 1B–D). Duplication and tandem fusion of an ancestral, bacterial-like *pfk* gene means that the eukaryotic genes are approximately twice as long [28,29]. Subsequent gene duplications in different eukaryotic lineages have also given rise to homologous subunit types and/or tissue-specific isoforms. For example, most yeast PFKs are hetero-oligomers of homologous α and β subunits, often active as α_4_β_4_ octamers [30]. Mammals have liver-, muscle- and platelet-specific PFK isoforms (PFKL, PFKM and PFKP, respectively), with subunits forming homo- and hetero-oligomers that include tetramers and higher order assemblies [30,31]. And a whole genome duplication in *Quercus rubra*, the northern red oak, gave rise to three pairs of *pfk* paralogues [32]. Finally, an additional gene fusion event occurred during the evolution of yeast PFKs, meaning that the enzymes from *Saccharomyces cerevisiae* (*Sce*PFK) and *Komagataella* (formerly *Pichia*) *pastoris* (*Kpa*PFK) have an extra N-terminal domain [29]. X-ray crystallography revealed this domain to be a distant structural relative of glyoxalase I in *Kpa*PFK [33].

In terms of surveying this sequence diversity, a key early study performed phylogenetic analyses using 152 PFK proteins [22]. The authors defined eight monophyletic sequence groups. A more recent study sought to place the 11 *Trichomonas vaginalis* PFKs within their phylogenetic context [25]. A tree constructed from the *T. vaginalis* PFKs, plus another 180 diverse sequences, now revealed 12 groups, albeit in a broadly similar topology to the earlier work [22].

Together, these two studies emphasised the complex evolutionary history of the Phosphofructokinase Superfamily. They uncovered monophyletic groups containing both ATP-dependent and PP_i_-dependent PFKs. This is consistent with multiple independent evolutionary events that led to changes in the phosphate donor, in different lineages [22,25,34]. Horizontal gene transfer has also been pervasive. In general, the phylogeny of PFKs does not match the phylogeny of the species in which they are found. For example, five of the eight sequence groups defined by Bapteste et al. [22] contained PFKs from prokaryotes and eukaryotes and there was evidence for transfer both to, and from, bacteria.

The complicated patterns of *pfk* evolution tend to obfuscate classifications in sequence databases. While the homologous superfamily is well defined as IPR035966 in InterPro, grouping into smaller sequence-based families paints a confusing picture. In InterPro, the Phosphofructokinase Superfamily is currently subdivided into 13 families (Table 1). However, the names of these families are overlapping, and it is difficult to understand what distinguishes any given family. For example, there is an InterPro family named ‘ATP-dependent 6-phosphofructokinase, prokaryotic-type’, and another named ‘ATP-dependent 6-phosphofructokinase, prokaryotic’ (Table 1). Both families contain many of the same proteins. In a similar vein, InterPro family IPR022953 is named ‘ATP-dependent 6-phosphofructokinase’ but contains both ATP-dependent and PP_i_-dependent PFKs. And family IPR012004 is named ‘Pyrophosphate-dependent phosphofructokinase TP0108-type’ but also contains ATP-dependent PFKs.

**Table 1:** InterPro families nested within the Phosphofructokinase Superfamily (IPR035966).

InterPro family	Family name
IPR022953	ATP-dependent 6-phosphofructokinase
IPR012003	ATP-dependent 6-phosphofructokinase, prokaryotic type
IPR012828	ATP-dependent 6-phosphofructokinase, prokaryotic
IPR009161	ATP-dependent 6-phosphofructokinase, eukaryotic type
IPR041914	ATP-dependent 6-phosphofructokinase, vertebrate type
IPR012004	Pyrophosphate-dependent phosphofructokinase TP0108 type
IPR050929	Phosphofructokinase type A
IPR011183	Pyrophosphate-dependent phosphofructokinase PfpB
IPR011404	Pyrophosphate–fructose 6-phosphate 1-phosphotransferase
IPR012829	Phosphofructokinase mixed-substrate PFK group III
IPR054846	PP_i_-dependent phosphofructokinase
IPR011405	Pyrophosphate-dependent phosphofructokinase SMc01852 type
IPR011403	Pyrophosphate-dependent phosphofructokinase TM0289 type

In summary, it is clear that names, sequences and genome annotations can be murky in the world of PFKs. As more PFK sequences are discovered, the boundaries between previously defined groups are tending to become blurrier. It is increasingly difficult to classify PFKs based on sequence alone. The most meaningful classifications are likely to be based on structural and functional features, as discussed in the following sections.

### Diversity in function, regulation and physiological roles

Hundreds of PFKs have been biochemically characterised in the past nine decades. At the time of writing, the BRENDA database [27] contained information on ATP-dependent PFKs (EC 2.7.1.11) from 180 different species, as well as PP_i_-dependent PFKs (EC 2.7.1.90) from 108 species. We used BRENDA as a starting point to review the range of steady-state kinetics parameters reported for members of the Phosphofructokinase Superfamily. We inspected the entries in BRENDA to identify wild-type enzymes that had been assayed for the formation of FBP, from F6P and the predominant phosphate donor (Mg^2+^-ATP for EC 2.7.1.11; Mg^2+^-PP_i_ for EC 2.7.1.90), under physiologically relevant conditions. For the ATP-dependent enzymes showing cooperative kinetics, Michaelis constants (*K*_M_ values) were often determined in the presence of an activator such as AMP [35], thus yielding hyperbolic saturation kinetics. Overall, this snapshot revealed that the ranges for turnover number (*k*_cat_) and for the two *K*_M_ values (F6P and the phosphate donor) are remarkably similar for ATP- and PP_i_-dependent PFKs (Table 2). The median *k*_cat_ value in BRENDA was ~110 s^-1^ for each class of PFK. The median *K*_M_ values for F6P were both *K*_M_^F6P^ = 0.3 mM. With respect to the phosphate donors, the median *K*_M_^ATP^ was ~0.05 mM and the median *K*_M_^PPi^ was ~0.03 mM. In 2018, a global survey of all wild-type enzymes in BRENDA revealed a median *k*_cat_ of ~10 s^-1^ and a median *K*_M_ of ~0.14 mM [46]. Thus, compared with other enzymes, PFKs tend to have higher-than-average turnover numbers and average Michaelis constants.

**Table 2:** Indicative steady-state kinetic parameters for wild-type members of the Phosphofructokinase Superfamily catalysing the formation of FBP from F6P.

Parameter	Value	Enzyme source	Reference
ATP-dependent PFKs			
Low *k*_cat_	49 s^-1^	*Escherichia coli*	[36]
High *k*_cat_	360 s^-1^	*Homo sapiens* (muscle isoform)	[37]
Low *K*_M_^F6P^	0.016 mM	*Ricinus communis* (castor bean)	[38]
High *K*_M_^F6P^	7 mM	*Fasciola hepatica* (liver fluke)	[35]
Low *K*_M_^ATP^	0.005 mM	*Cucumis sativus* (cucumber)	[39]
High *K*_M_^ATP^	0.82 mM	*Bacillus methanolicus*	[40]
PP_i_-dependent PFKs			
Low *k*_cat_	16 s^-1^	*Methylobacterium nodulans*	[24]
High *k*_cat_	330 s^-1^	*Entamoeba histolytica*	[41]
Low *K*_M_^F6P^	0.01 mM	*Naegleria fowleri*	[42]
High *K*_M_^F6P^	3.8 mM	*Oryza sativa* (rice)	[43]
Low *K*_M_^PPi^	0.005 mM	*Propionibacterium freudenreichii*	[44]
High *K*_M_^PPi^	0.58 mM	*Sanseviera trifasciata* (snake plant)	[45]

Allosteric regulation also varies significantly across the Phosphofructokinase Superfamily. In general, the bacterial ATP-dependent PFKs are activated by ADP and inhibited by phosphoenolpyruvate [30]. Some bacterial ATP-dependent PFKs are also inhibited by PP_i_, often when the organism also possesses a PP_i_-dependent PFK [47]. However, the strength of this allosteric regulation can vary. For example, the enzyme from *Lactobacillus delbrueckii* is relatively insensitive to its effectors, ADP and phosphoenolpyruvate, with dissociation constants that are 2–3 orders of magnitude higher than the values for *Eco*PFK and *Gst*PFK [48].

Allosteric regulation in the eukaryotic ATP-dependent PFKs tends to be significantly more complex and was reviewed by Schöneberg et al. [30]. Over 20 effectors have been identified for various eukaryotic enzymes, with AMP and fructose 2,6-bisphosphate joining ADP as important physiological activators. ATP is both a substrate and an allosteric inhibitor, with metabolites such as citrate and lactate potentiating its inhibitory effect [49]. Recently, a powerful new mass spectrometry method was described for high-throughput discovery of protein-metabolite interactions [50]. This confirmed existing metabolite interactions of the human liver and platelet PFK isoforms, while also providing evidence for a dozen or so new interactions. These interactions—with metabolites such as 2,3-bisphosphoglycerate, CTP and 5-hydroxy-l-tryptophan—require further biochemical investigation; however, the implication is that more allosteric effectors remain to be discovered for the eukaryotic ATP-dependent PFKs.

In contrast with the ATP-dependent enzymes, many PP_i_-dependent PFKs—including all those characterised from bacteria—are non-allosteric. For example, the PFK from *Acetivibrio thermocellus* (formerly *Clostridium thermocellum*) was recently tested in the presence of 11 different metabolites at two different concentrations, and none had a significant effect on activity compared with the ATP-dependent PFK from the same organism [47]. However, fructose 2,6-bisphosphate activates the PP_i_-dependent PFKs from plants including rice, potato, banana and strawberry [43,51-53]. AMP was also shown to be an activator of the PP_i_-dependent PFK from the amoeba, *Naegleria fowleri* [42].

The identity of the phosphate donor has significant metabolic and physiological implications. ATP-dependent PFKs catalyse a reaction (F6P + ATP → FBP + ADP) that is far from equilibrium and therefore effectively irreversible *in vivo* (although its reverse has been assayed *in vitro*; [54]). Not only is the forward reaction highly favourable under standard conditions (ΔG°′ = –17.8 ± 1.3 kJ mol^-1^), but a further thermodynamic driving force comes from the intracellular (ATP)/(ADP) ratio, which is typically around 10 [55,56]. In contrast, the standard Gibbs free energy of PP_i_-dependent phosphotransfer is smaller (ΔG°′ = –4.6 ± 1.4 kJ mol^-1^) and the intracellular ratio of PP_i_ to P_i_ is typically around 0.1. This lower driving force means the glycolytic pathway lies closer to thermodynamic equilibrium in organisms with PP_i_-dependent PFKs [56]. As a result, PP_i_-dependent PFKs are often assayed in both the forward (F6P + PP_i_ → FBP + P_i_) and reverse (FBP + P_i_ → F6P+PP_i_) directions. Careful comparisons of the steady-state kinetic parameters in each direction have been carried out for the PP_i_-dependent PFKs from the unicellular eukaryote *Giardia lamblia* [57], and the bacteria *Methylobacterium nodulans* and *Methylosinus trichosporium* [24]. For all three enzymes, *k*_cat_ is identical (or near-identical) in the forward and reverse directions. However, each enzyme shows a lower *K*_M_ for the bisphosphorylated substrate, FBP, meaning that in each case the overall catalytic efficiency (*k*_cat_/*K*_M_) is 3- to 13-fold higher for the reverse reaction (Supplementary Table S1).

An interesting corollary is that organisms with a reversible, PP_i_-dependent PFK could use it for both the glycolytic forward reaction and the gluconeogenic reverse reaction *in vivo* [12,58]. This is in contrast with organisms with ATP-dependent PFKs, which employ a separate enzyme, fructose-1,6-bisphosphatase (FBPase), for hydrolysing FBP to F6P in gluconeogenesis. Thus, one might expect the gene for FBPase to be inactivated or lost from organisms with PP_i_-dependent PFKs. While PP_i_-dependent PFKs have been proposed to play this bifunctional role in *Amycolatopsis methanolica* [59] and *Porphyromonas gingivalis* [60], these anaerobic bacteria also contain the gene for a dedicated FBPase. It remains to be determined whether any organism uses a PP_i_-dependent PFK as its primary enzyme for both glycolysis and gluconeogenesis.

A third physiological role for PP_i_-dependent PFKs lies in a variation of the classical pentose phosphate pathway. Four decades ago, Reeves et al. discovered that *E. histolytica* lacks a transaldolase for catalysing sedoheptulose 7-phosphate + glyceraldehyde 3-phosphate → erythrose 4-phosphate + F6P. Instead, the *E. histolytica* PFK (*Ehi*PFK) phosphorylates the seven-carbon metabolite ᴅ-sedoheptulose 7-phosphate (S7P), forming sedoheptulose 1,7-bisphosphate (SBP). The resulting SBP can be cleaved into erythrose 4-phosphate and dihydroxyacetone phosphate by the glycolytic enzyme, fructose bisphosphate aldolase, effectively replacing the absent transaldolase [61]. More recently, this SBP pathway was also shown to operate in cellulolytic clostridia lacking transaldolase genes [62]. Metabolomics experiments confirmed a significant intracellular SBP pool in *Clostridium thermosuccinogenes*. The PP_i_-dependent PFKs from *C. thermosuccinogenes* and *A. thermocellus* were also purified and shown to be highly active. Their *k*_cat_/*K*_M_ values were > 10^6^ s^-1^ M^-1^ when F6P was the substrate, and reduced by only four- to six-fold with S7P [62].

Some PP_i_-dependent PFKs also have a fourth physiological role, which is to maintain PP_i_ homeostasis. Most organisms possess a pyrophosphatase (PPase) to hydrolyse PP_i_ and prevent its toxic accumulation. However, *A. thermocellus* does not contain a PPase and the gene for its PP_i_-dependent PFK could only be deleted when a gene for a PPase was also introduced [63]. Similarly, in the parasite *Toxoplasma gondii,* the PP_i_-dependent PFK—but not the ATP-dependent PFK—was shown to be essential for growth [64]. Depleting the PP_i_-dependent PFK led to increased PP_i_ levels and reduced protein synthesis by 40%. The fitness defect could be rescued by over-expressing PPase, demonstrating an essential role for the PP_i_-dependent PFK in maintaining PP_i_ homeostasis [64].

### A dive into the structural universe of ATP-dependent PFKs

At the time of writing, there are 45 structures of Phosphofructokinase Superfamily enzymes available in the Protein Data Bank (PDB) [65]. We have compiled basic information about each of these structures in Supplementary Table S2. Perhaps surprisingly—given the central role of PFK in glycolysis and its rich history of biochemical research—the 45 structures are from only 14 different organisms. Forty of the structures are ATP-dependent PFKs and only five are PP_i_-dependent PFKs.

**Figure 3:**
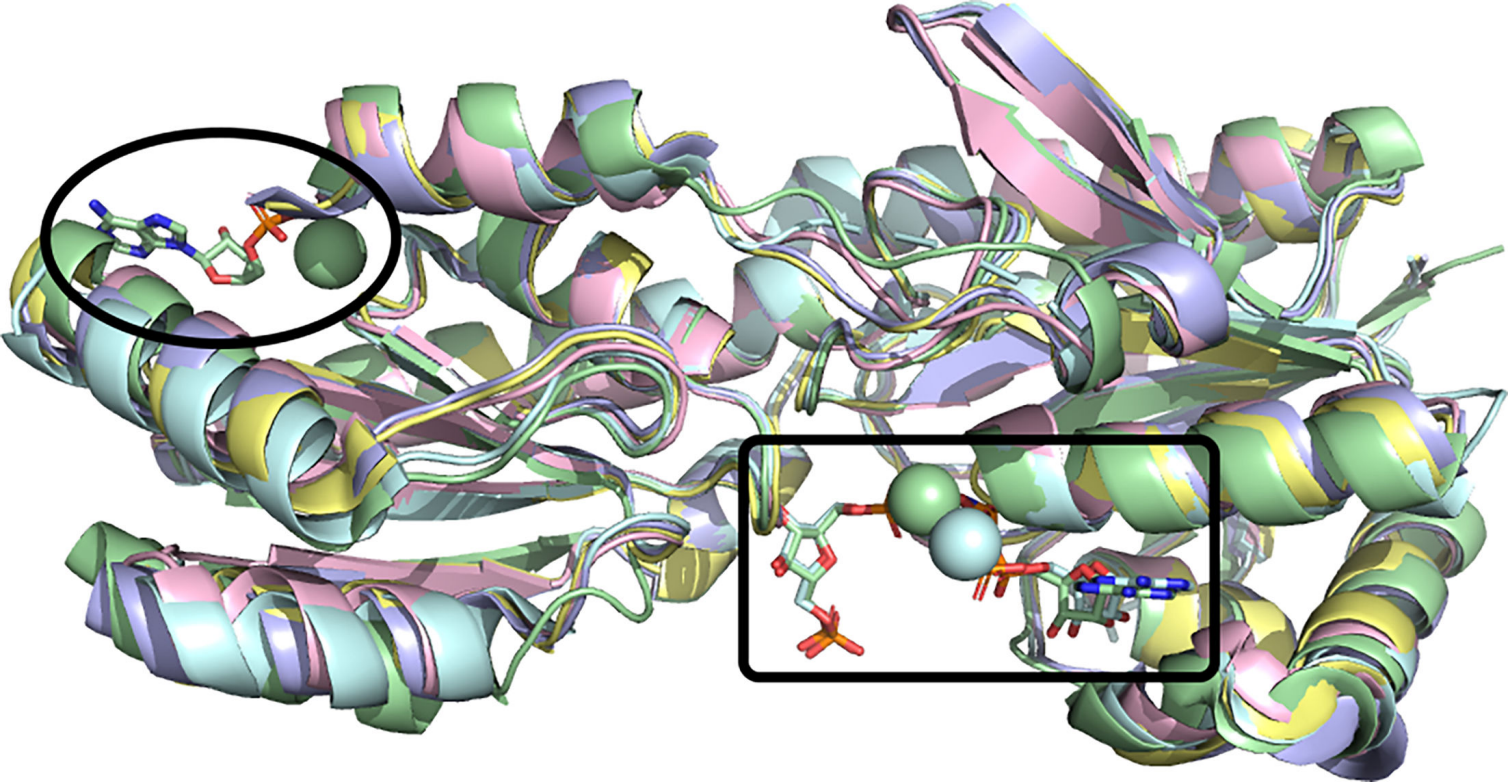
Many bacterial PFKs have near-identical structures. Structures of the ATP-dependent PFKs from five bacterial species have been determined. Representative subunits from each of these are shown superimposed. The rectangle highlights the active site and the oval highlights the effector site. PFKs, 6-phosphofructokinases.

Within the set of ATP-dependent PFKs, 18 structures are from five species of bacteria and they all have the same architecture first glimpsed by Evans et al. [5,7]. Each enzyme is active as a homotetramer, with subunits of 319 to 330 residues in the different species. The subunit structures from each species are near-identical, with an average RMSD between any two chains of approximately 0.6 Å (Figure 3). *Gst*PFK has remained a particularly important exemplar, with eight structures in the PDB. These have confirmed that the physiologically relevant allosteric regulators, ADP and phosphoenolpyruvate, occupy the same effector site in the R state and T state, respectively [6,10,66].

The second major group of ATP-dependent PFK structures is from the yeasts *S. cerevisiae* and *K. pastoris*, and the mammals *Oryctolagus cuniculus* (rabbit) and *Homo sapiens*. There are 16 PFK structures from these eukaryotes in the PDB (Supplementary Table S2). As a result of the tandem duplication in their evolutionary history (*vide supra*), their subunits are 750–1000 residues in length. The detailed molecular architecture of these enzymes was first revealed in 2011, with near-simultaneous publication of the *Kpa*PFK structure [33] and then the *S. cerevisiae* and rabbit muscle structures [67]. The latter structure was a homodimer, rather than the physiologically active homotetramer. On the other hand, the yeast enzymes were determined in active, heterooligomeric forms. *Sce*PFK contained two α subunits and two β subunits [67]. Most impressively, the structure of *Kpa*PFK also contains a third subunit, γ, and was solved as a (αβγ)_4_ dodecamer of almost 1 MDa [33]. The structure also revealed that the γ subunit had most likely evolved from an ancient *S*-adenosylmethionine-dependent methyltransferase.

The first low-resolution structure of a human PFK (PFKM at 6 Å resolution) was reported in 2014 [68], followed by higher resolution PFKP structures the following year [69,70]. The structure of the human liver isoform, PFKL, was the last of this group to be reported and also the first PFK to be determined by cryoEM rather than X-ray crystallography [71].

**Figure 4:**
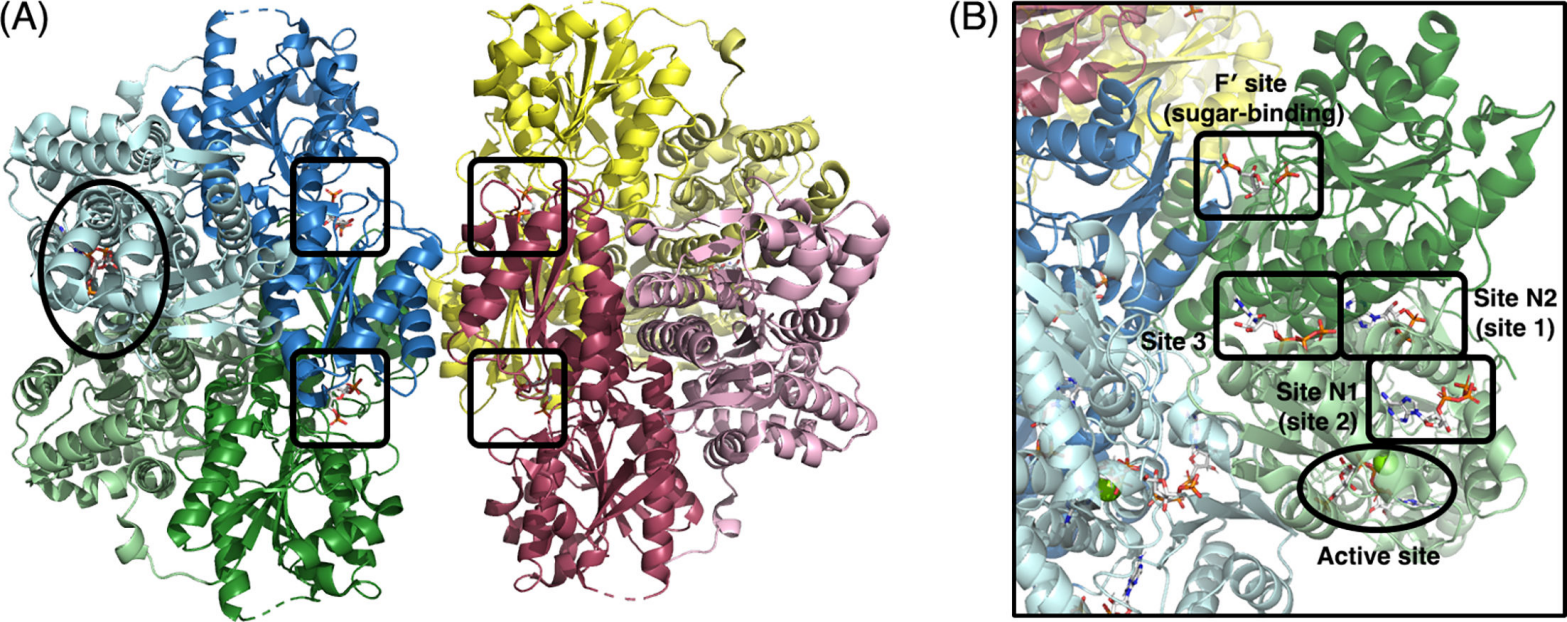
The complex architecture of some eukaryotic ATP-dependent PFKs. (**A**) The human PFKP enzyme (PDB ID: 4XZ2, [69]). The four chains in the homotetramer are coloured blue, green, yellow and red. The lighter shaded part of each subunit is the N-terminal half containing the active site. The darker shaded part is the C-terminal (regulatory) half. The active site in the blue subunit is highlighted with an oval. The four F´ regulatory sites (boxed) are on the inner surface of the tetramer and occupied by fructose-1,6-bisphosphate in this structure. (**B**) Locations of the effector sites in human PFKL [72]. The image shows the T state structure (PDB ID: 8W2H). Chains are coloured as described for panel (**A**). The oval shows the position of an active site. The rectangles indicate effector sites and are labelled according to the two common naming conventions [69,72]. F6P in the active site and ADP in effector site N2 were positioned by overlaying the R state structure (PDB ID: 8W2G). PFK, 6-phosphofructokinase.

Other than the accessory γ subunit of *Kpa*PFK, each subunit of the yeast and mammalian PFKs contains four Rossmann folds (Figure 4A). The two N-terminal Rossmann folds contain the active site. Binding modes for the substrates, F6P and ATP, have been retained from the bacterial ATP-dependent enzymes [33,67]. On the other hand, catalytic activity has been lost from the C-terminal half of each monomer. Instead, this part of the structure is now important for stability [73] and for the complex allosteric regulation of these enzymes (Figure 4B). Schöneberg et al. have written an excellent review of the allosteric effector sites [30], to which the interested reader is referred for more detail. In brief, at the former C-terminal active site the ATP-binding pocket has been lost entirely, while the F6P-binding pocket (the F' site) has evolved to become the sugar-binding effector site that is important for activation by fructose 2,6-bisphosphate (Figure 4A). Two new effector sites have evolved in the yeast and mammalian enzymes, which have no counterparts in the bacterial PFKs (Figure 4B). The first of these, site N1 (also named site 2 in PFKL; [72]) binds ATP as an allosteric inhibitor [33,67]. The second novel site N2 (named site 1 in PFKL) is an activation site that binds ADP [67,72].

Until recently, no yeast or mammalian PFK had been visualised in both the R state and the T state. Therefore, it was an important advance when Lynch et al. used cryoEM to determine structures of human PFKL in both states, elucidating key details of the R to T transition [72]. As with the R-to-T transition in the bacterial enzyme [10], a 7° rotation disrupts the F6P-binding part of the active site. However, this rotation is between PFKL monomers and along a different axis than the conformational change in *Gst*PFK.

Critically, the T state PFKL structure captured ATP in effector site 3 (Figure 4B), which is in an equivalent location to the effector site in *Gst*PFK. Compared with the PFKL R state (in which site 3 was unoccupied), ATP binding led to localised unfolding of an α helix and shifted the positions of two arginine residues (Arg201 and Arg292). In the R state, these residues interact with the phosphate of the substrate, F6P, in an adjoining active site [72]. Thus, ATP at site 3 is an important inhibitory signal that contributes to disrupting the F6P binding pocket. Furthermore, a phosphate occupies site 3 in previously determined R state PFKP structures [69,70]. Because phosphate ions have been shown to increase PFK activity [74], Lynch et al. suggested that this effector site may play dual activating and inhibitory roles—akin to the single effector site in *Gst*PFK [72].

An interesting corollary is to ask how *Gst*PFK binds ADP to maintain the R state, whereas PFKL binds ATP to maintain the T state. Overlaying the two enzymes reveals quite different binding poses for the two effector molecules. While the phosphates of the two effectors occupy equivalent positions, the adenosine moiety in PFKL is rotated by approximately 180° compared with its position in the *Gst*PFK effector site (Supplementary Figure S1). Thus, the location of the effector site has been conserved through evolution, but the specific interactions made by the bacterial and eukaryotic enzymes have diverged.

The R and T state structures of PFKL have also helped to resolve the role of a C-terminal tail of approximately 20 residues. Deleting this tail was known to lock PFKP in an active conformation [69]. In the PFKL structures, it was disordered in the R state, but in the T state, it adopted an extended conformation that bridged across the regulatory and catalytic domains [72]. Molecular dynamics simulations confirmed that this positioning of the tail stabilises the T state [72]. Interestingly, the C-terminus is also known to become phosphorylated when PFKL is activated in response to insulin signalling [75]. This phosphorylation may displace the tail and favour transition to the active R state [72].

Overall, it is clear that the tandem duplication associated with these eukaryotic PFKs has potentiated the evolution of sophisticated regulatory mechanisms and that future work will continue to reveal more details of these.

### *Trypanosoma brucei* PFK: an unusual structure makes a novel drug target

In addition to the enzymes described above, another well-characterised, eukaryotic, ATP-dependent PFK is from the protozoan that causes African sleeping sickness, *Trypanosoma brucei* [76]. The *T. brucei* PFK (*Tbr*PFK) was the first eukaryotic PFK to have its structure determined [77], and there are now six structures available in the PDB (Supplementary Table S2). It is an outlier because it is not a product of the gene duplication that characterises other eukaryotic ATP-dependent PFKs. Instead, it is closer in sequence to the PP_i_-dependent PFKs [78]. With 487-residue subunits, it also contains extra elements of structure that set it apart from shorter bacterial ATP-dependent PFKs. These structural decorations include a novel N-terminal domain and an extra 20-residue loop that is also found in some PP_i_-dependent PFKs [77]. The ATP-bound structure of the *Tbr*PFK dimer-of-dimers also revealed a C-terminal extension dubbed the ‘reaching arm’, which adopts a long α-helical conformation, extends across the inter-dimer interface, and forms a lid over the effector binding site [34]. The only known allosteric activator of *Tbr*PFK is AMP, which binds at the same effector site found in ATP-dependent bacterial PFKs [79]. However, AMP is rotated by 180° compared with ADP occupying the equivalent site in *Eco*PFK.

**Figure 5:**
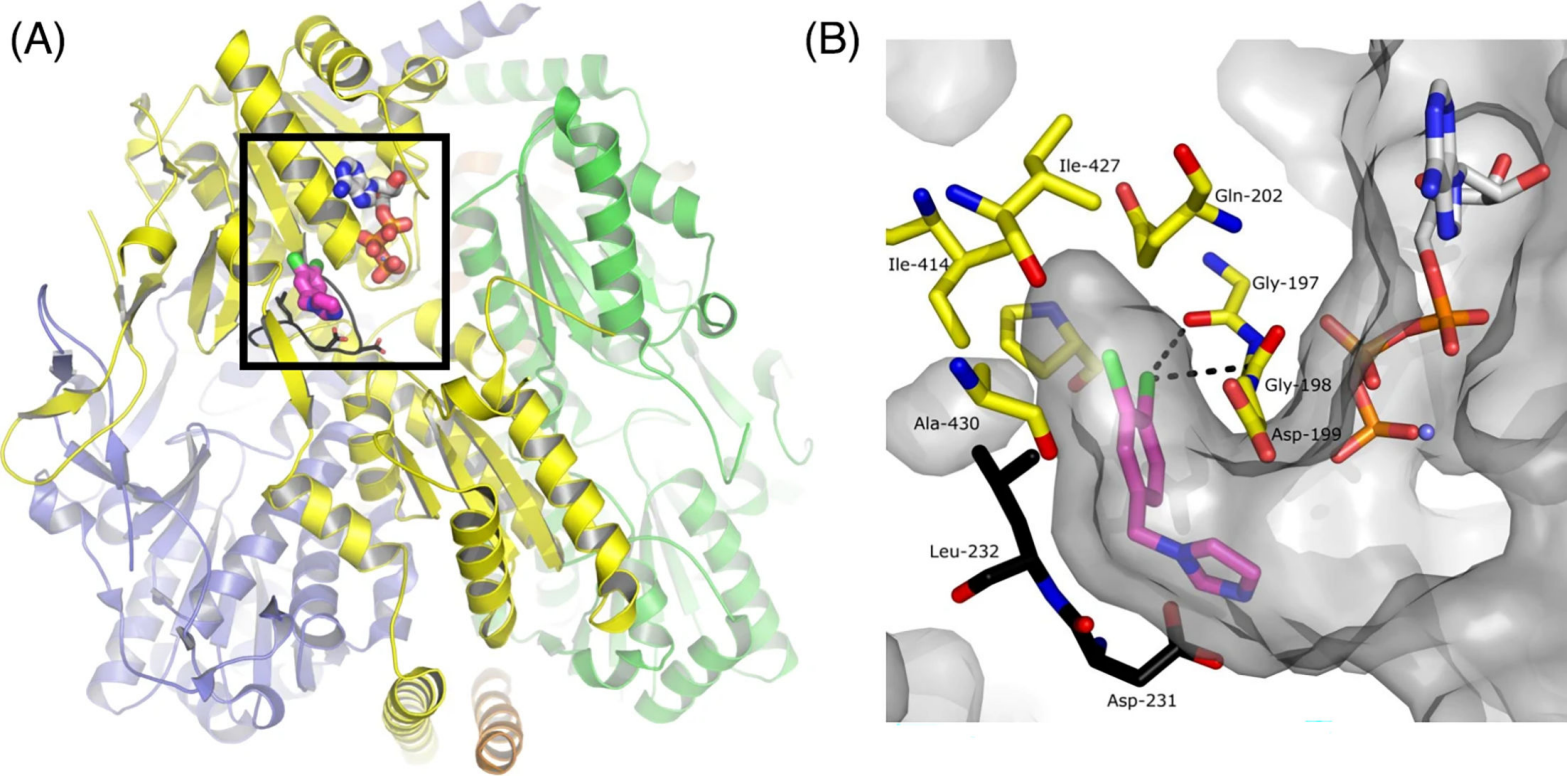
Structure-based design of *Tbr*PFK inhibitors that cure sleeping sickness in mice. (**A**) The *Tbr*PFK homotetramer, with chains coloured yellow, green, purple and orange. The box shows the location of an active site. The structure (PDB ID: 6QU5) contains ATP, displayed as thick sticks. The dichlorophenyl lead compound, CTCB-12, is shown in pink, with its chlorine atoms in green, in the adjacent allosteric pocket. (**B**) Zoomed-in view of the boxed region in (**A**) showing the dichlorophenyl ring of CTCB-12 occupying the allosteric pocket. Two key residues from the mobile activating loop, Asp231 and Leu232, are in black. By blocking the movement of Leu232 into the allosteric pocket, CTCB-12 prevents the mobile loop from adopting its R state conformation in which Asp231, as well as the catalytic base Asp229, are positioned for catalysis. The figure is reproduced from reference [81] under a Creative Commons Attribution 4.0 International License (CC BY 4.0; https://creativecommons.org/licenses/by/4.0/). PFK, 6-phosphofructokinase.

The glycolytic enzymes of *T. brucei* have attracted attention as drug targets because the form of the parasite in the bloodstream of infected humans is wholly dependent on glycolysis for producing ATP [80]. Recently, the unusual structural features of *Tbr*PFK were used to guide a noteworthy drug discovery campaign [81]. McNae et al. developed a series of allosteric inhibitors that do not compete with ATP binding in the active site but do prevent a mobile loop from adopting the R state conformation (Figure 5). The inhibitors showed IC_50_ values as low as 30 nM for inhibiting *Tbr*PFK. Most impressively, the three best compounds had faster parasite kill times than existing drugs in the clinic, did not significantly inhibit human PFKs *in vitro* and rapidly cleared parasites in a mouse model of *T. brucei* infection [81].

### The PP_i_-dependent PFKs: decorated structures and insights into phosphate donor specificity

There are currently five structures of PP_i_-dependent PFKs available in the PDB (Supplementary Table S2). Four are from bacteria: *Borrelia burgdorferi* (two structures); *Nitrosospira multiformis*; and *Marinobacter aquaeolei*. The fifth is one of the PFK isoforms from the protozoan, *T. vaginalis*. Of these, just one of the *B. burgdorferi* PFK (*Bbu*PFK) structures has an associated publication [13], although the *T. vaginalis* structure (*Tva*PFK) was only deposited in late 2024. The PP_i_-dependent PFKs have the two-Rossmann architecture of their ATP-dependent bacterial homologues. However, a variety of insertions and extensions mean that their subunits are also larger than those shown in Figure 3.

**Figure 6:**
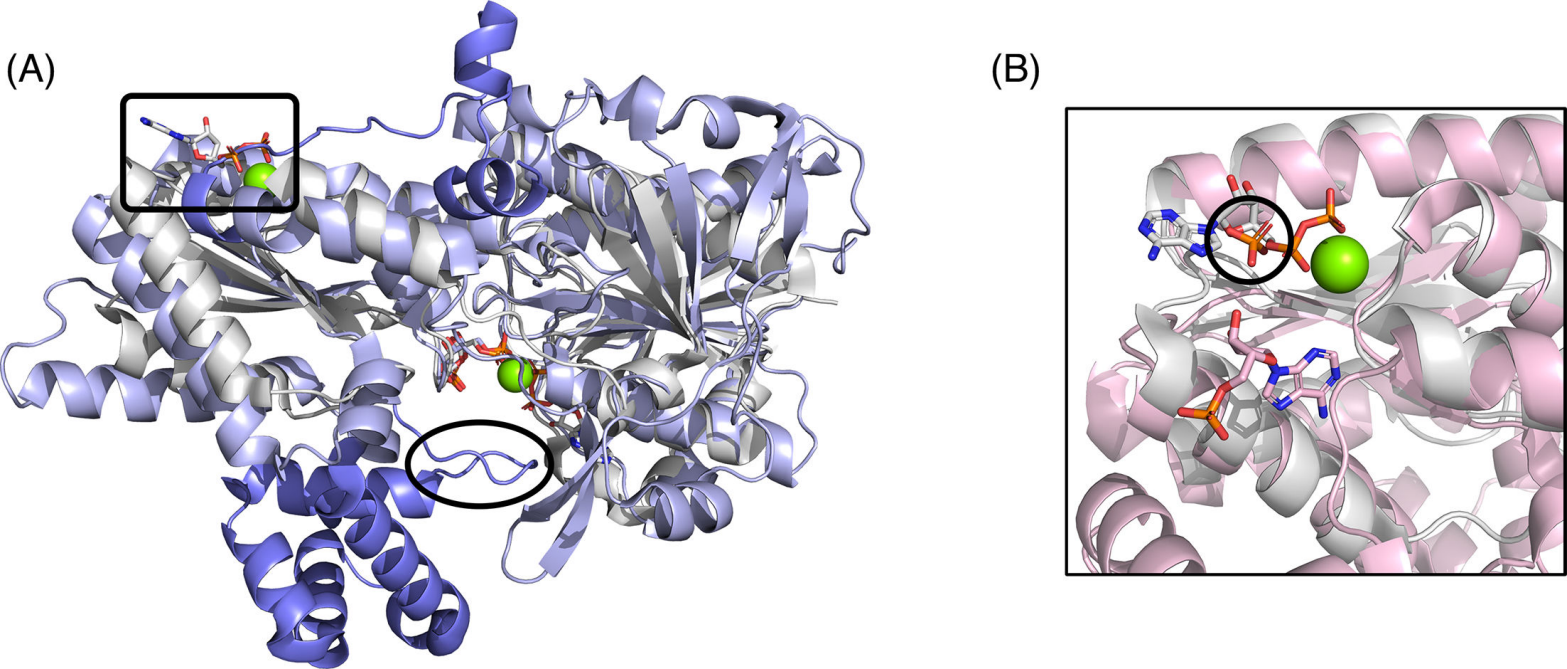
Superposition of PP_i_-dependent PFKs with the ATP-dependent *E. coli* enzyme. (**A**) A protomer of *Bbu*PFK (PDB ID: 2F48) in purple, aligned with *Eco*PFK (PDB ID: 1PFK) in grey. In *Bbu*PFK, the C-terminal extension and the helical domain insertion are shown in a darker shade. The effector site in *Eco*PFK, with ADP and Mg^2+^ bound, is indicated by the rectangle. This site is disrupted by the C-terminal extension in *Bbu*PFK. The β hairpin from the *Bbu*PFK domain insertion, which closes over the active site, is shown in the oval. (**B**) Superposition of *Tva*PFK (PDB ID: 9DQM) in pink with *Eco*PFK (PDB ID: 1PFK) in grey, in the region of the effector site. The *Tva*PFK structure has AMP (pink sticks) bound in a putative effector site that is distinct from the site where ADP (white sticks) binds in *Eco*PFK. The *Tva*PFK structure also includes a bound phosphate (circled) that overlays the α-phosphate of ADP in the *Eco*PFK structure. PFK, 6-phosphofructokinase; PPi, inorganic pyrophosphate.

The landmark study on *Bbu*PFK, the enzyme from the Lyme disease pathogen, revealed a highly decorated homodimer of 555-residue subunits [13]. The two Rossmann-containing domains adopt a highly similar structure to *Eco*PFK (RMSD = 1.2 Å). However, *Bbu*PFK has extensions of 70 residues at the N-terminus and 34 residues at the C-terminus, with the latter structural element disrupting the effector binding site seen in *Eco*PFK (Figure 6A). *Bbu*PFK also contains an 83-residue insertion that forms a novel domain, comprising four α helices and a β hairpin that is positioned over the active site (Figure 6A). The β hairpin plays a role in conferring PP_i_ specificity, with the side chain of His384 positioned such that it would block binding of an ATP ribose group [13].

The *Bbu*PFK structure also shed light on previous sequence analyses [19] and mutagenesis experiments [82], which had identified putative signatures of phosphate donor specificity (Supplementary Table S3). The first of these is an invariant glycine in ATP-dependent PFKs, found in the motif GGDG. *Bbu*PFK and other PP_i_-dependent PFKs possess GGDD instead. The negatively charged aspartate that differs between the two (Asp177 in *Bbu*PFK) occupies the site of the ATP α-phosphate. Not only does this prevent ATP from binding, but it also ensures that PP_i_ binds in the correct register; that is, it binds in a position corresponding to the β- and γ-phosphates of ATP [13]. Secondly, in *Bbu*PFK, Lys203 is found in the PP_i_-PFK signature motif, PKTIDXD. Glycine usually replaces the lysine in ATP-dependent PFKs [82]. In the solved structure of *Bbu*PFK, Lys203 makes a salt bridge with the sulfate in the active site that mimics one of the phosphates of PP_i_. More specifically, the sulfate occupies the position where the β-phosphate of ATP would be found in an ATP-dependent PFK [13]. In an unpublished *Bbu*PFK structure (PDB ID: 2F48), the lysine is instead 3.1 Å from the phosphate at the 1-position of the bound product, FBP. This appears to confirm a role for Lys203 in positioning PP_i_ and also in aiding with polarisation of the phosphoanhydride bond that is cleaved during catalysis [13]. Interestingly, the ‘PP_i_-like’ ATP-dependent enzyme, *Tbr*PFK, also has a lysine in this position (Supplementary Table S3), showing that the P(G/K)TIDXD motif is not, *a priori*, an indicator of phosphate donor specificity [34]. Finally, the *Bbu*PFK structure highlighted the role of Asn181 in sterically preventing the adenine moiety of ATP from binding in the active site [13]. In all solved structures of ATP-dependent PFKs, the equivalent residue is a glycine. While it is not conserved as asparagine in PP_i_-dependent PFKs, it is always larger than glycine (Supplementary Table S3).

*Tva*PFK (denoted TvPP_i_-PFK1 in [25]) was first purified over 25 years ago and is a homotetramer of 434-residue subunits [19]. While a full description of its structure (PDB ID: 9DQM) must necessarily await an accompanying publication, it is tantalising to note that this eukaryotic PP_i_-dependent enzyme contains extra helices in the FBP-binding domain. These contribute to a binding site that is occupied by AMP in the deposited structure (Figure 6B) and which appears to be in a similar location to site N1 in the eukaryotic ATP-dependent enzymes (Figure 4B). Further, an inorganic phosphate in the *Tva*PFK structure occupies the same position as the α-phosphate of ADP in the bacterial effector site (Figure 6B), perhaps making it equivalent to site 3 in human PFKL (Figure 4B).

### The nature of the ancestral PFK

In the sections above, we summarised the enormous diversity in phosphate donor specificity, reaction specificity, allosteric regulation and structural innovations in the Phosphofructokinase Superfamily, across the tree of life. A longstanding question therefore concerns the nature of the superfamily’s common ancestor [12,58,83]. ATP-dependent bacterial enzymes such as *Eco*PFK have the smallest subunits of the known PFKs, and in their classic phylogenetic study, Bapteste et al. used parsimony to conclude that the ancestral PFK was also ATP-dependent [22]. On the other hand, *Eco*PFK is allosterically regulated, and allostery is a derived rather than ancestral trait [84]. Thus, others have hypothesised that the ancestral PFK was non-allosteric and PP_i_-dependent [20,85], despite the structural decorations on modern-day enzymes such as *Bbu*PFK. Of note, the non-allosteric PP_i_-dependent PFKs from the archaeon, *Thermoproteus tenax*, and the bacterium, *Dictyoglomus thermophilum*, are minimally sized homodimers that are approximately the same size as *Eco*PFK (337 and 345 residues per subunit, *versus* 319 for *Eco*PFK), and may represent descendants of the most ancient lineage [20,86].

In eukaryotes, there is strong evidence for frequent phosphate donor switching during evolution. Activity with ATP was introduced into the PP_i_-dependent *Ehi*PFK by a single point mutation, showing that this enzyme contains a latent ATP-binding site [82]. In the other direction, sequence and structure analyses have shown that the ATP-dependent *Tbr*PFK evolved from a PP_i_-dependent ancestor [34]. However, it is useful to recall that eukaryotes only arose about two billion years ago [87]; by the time they appeared, prokaryotes had already been evolving for over two billion years [88].

Glycolysis and gluconeogenesis are among the most ancient metabolic pathways on Earth, and they originated when all life was strictly anaerobic [89,90]. The phylogenetic distribution of extant PP_i_-dependent PFKs—across eukaryotes, bacteria and archaea—also correlates with an anaerobic lifestyle. Having this type of PFK is beneficial in fermentative metabolism because, compared with ATP-dependent PFKs, PP_i_-dependent PFKs deliver a net yield of one extra ATP per molecule of glucose in glycolysis [58]. In the primordial world, pyrophosphate was also likely to have been present as an essential component of energy metabolism [91]. Thus, in this ancient anaerobic environment, there is likely to have been a strong selective advantage for a PP_i_-dependent PFK.

Finally, the first organisms had small genomes and, consequently, restricted enzyme repertoires [92]. A widely accepted model posits a patchwork metabolism in these cells, with multifunctional enzymes catalysing reactions in multiple pathways [93,94]. As discussed above, PP_i_-dependent PFKs catalyse phosphotransfer in the gluconeogenic direction (forming F6P and PP_i_) with similar efficiency to the glycolytic reaction (Supplementary Table S1). Not only that, but gluconeogenesis is thought to have evolved before glycolysis [95], and some PP_i_-dependent PFKs functionally replace the transaldolase in another ancient branch of metabolism, the pentose phosphate pathway [62]. Thus, it is conceivable that a primordial cell made use of one multifunctional, PP_i_-dependent enzyme in three physiological roles.

On balance, it is the non-allosteric, multifunctional, PP_i_-dependent PFKs that possess an array of ancestor-like characteristics. However, as discussed in the previous section, the number of PP_i_-dependent PFK structures is currently low. Further exploration of ancestor-like PP_i_-dependent PFKs will undoubtedly generate new hypotheses on plausible evolutionary routes to ATP dependence, allostery and narrowed substrate range.

### Future directions: diversity, dynamics, diseases and drugs

Within the Phosphofructokinase Superfamily, hundreds of functional parameters and dozens of structures are now available in BRENDA and the PDB, respectively. At the same time, there are still important unanswered questions and exciting opportunities for future research.

We have highlighted the taxonomic bias in our structural knowledge of PFKs, which mirrors the bias for all other enzymes [96]. For example, no superfamily members from plants or archaea have had their structures determined. Similarly, the available bacterial structures are from only three of the 175 recognised phyla in the Genome Taxonomy Database (release 220; [97]). Given the structural diversity that has already been described from 14 (taxonomically non-representative) species, it seems certain that new structures will continue to reveal domain fusions, insertions and subunit compositions that underpin complex regulatory behaviour and, ultimately, the adaptation of organisms to their environment.

While structural biology has been essential for elucidating the basis of the R-to-T allosteric transition, a noteworthy recent advance was the use of molecular dynamics to aid with understanding this transition in human PFKP and PFKL [72,98]. Computational approaches for identifying the networks of residues in proteins that transmit allosteric signals are advancing rapidly [99]. Combined with increasingly powerful molecular dynamics simulations [100], an exciting opportunity now exists to understand the allosteric transition at hitherto unseen levels of spatial and temporal resolution. Moreover, with atomistic understanding comes programmability. In the era of synthetic biology, there is significant interest in designing allosterically switchable properties into proteins [101,102]. Much of metabolic engineering involves programming carbon flux. There is considerable potential to build on groundbreaking work [56] by designing exactly the right PFK for the desired outcome in microbial cell factories.

Finally, our increasing knowledge of PFK biochemistry will likely translate into the clinic. As discussed above, *Tbr*PFK has already been validated as a target for promising anti-trypanosome drugs [81]. In humans, Tarui disease (also known as glycogen storage disease type VII) is a rare autosomal recessive disorder associated with the absence of PFK activity in skeletal muscle [103,104]. Structures of the human isoforms are rationalising disease-causing mutations [69]. As an important regulator of glycolytic flux, PFK has also attracted attention because glucose metabolism is dysregulated in a range of conditions including cancer, diabetes and Alzheimer’s disease [98,105]. Determining the structure of human PFKP enabled 44 somatic mutations from cancers to be mapped onto it, leading to the prediction that 28 of these would affect activity, three of which were validated experimentally [70]. One specific example is the PFKP mutation D564N—identified in colon cancer—which was shown to decrease enzyme activity but was proposed to favour cancer cell survival in tumour microenvironments where oxidative stress is present [98]. On the other hand, up-regulation of PFKL expression is directly connected to the Warburg effect, in which cancerous cells increase glycolysis independent of the oxygen level [106]. This has recently been explored in hepatocellular carcinoma (HCC), in which the E3 ubiquitin ligase A20 usually acts as a tumour suppressor by targeting PFKL for degradation. Malignant transformation results in reduced expression of A20, which in turn causes PFKL to accumulate. This facilitates cell proliferation and migration, HCC growth and reduced patient survival [107].

Future therapeutic approaches may therefore lie in designing small molecules that either activate or deactivate PFK, depending on the particular disease. For example, a small molecule, NA-11, inhibited the pro-inflammatory oxidative burst in neutrophils specifically by binding at effector site N2 in PFKL (Figure 4B) and activating the enzyme [71]. The mechanism of action suggested a wholly novel approach to controlling inflammatory disorders such as acute respiratory distress syndrome, and potentially treating other autoimmune diseases. On the other hand, a drug repurposing screen identified an antipsychotic medication, penfluridol, that specifically inhibited PFKL in oesophageal squamous cell carcinoma, suppressing tumour growth and inducing apoptosis [108].

Our biochemical knowledge of the Phosphofructokinase Superfamily has come a long way in the past 90 years. Nevertheless, the diversity of structures and functions across the tree of life and the complex role of PFK at the heart of the metabolic network ensure that much still remains to be discovered.

## Supplementary material

online supplementary figure 1

## Abbreviations

BRENDA: BRaunschweig ENzyme DAtabase
EC: Enzyme Commission
FBP: fructose 1,6-bisphosphate
FBPase: fructose 1,6-bisphosphatase
F6P: fructose 6-phosphate
PDB: Protein Data Bank
PFK: 6-phosphofructokinase
PFKL: liver isoform of mammalian phosphofructokinase
PFKM: muscle isoform of mammalian phosphofructokinase
PFKP: platelet isoform of mammalian phosphofructokinase
SBP: sedoheptulose 1,7-bisphosphate
S7P: sedoheptulose 7-phosphate
