# Supplementary material for: The more we learn, the more diverse it gets: structures, functions and evolution in the Phosphofructokinase Superfamily
Source: Biochem J. 2025 May 6;482(9):467–83. doi: 10.1042/BCJ20253024 (PMC12203962; doi:10.1042/BCJ20253024)
Supplement: online supplementary figure 1 [file BCJ-482-09-BCJ20253024-s001.pdf]

Supplementary Material for:

The more we learn, the more diverse it gets: structures, functions and evolution in the Phosphofructokinase Superfamily

Jordan A. Compton & Wayne M. Patrick

Supplementary Figure

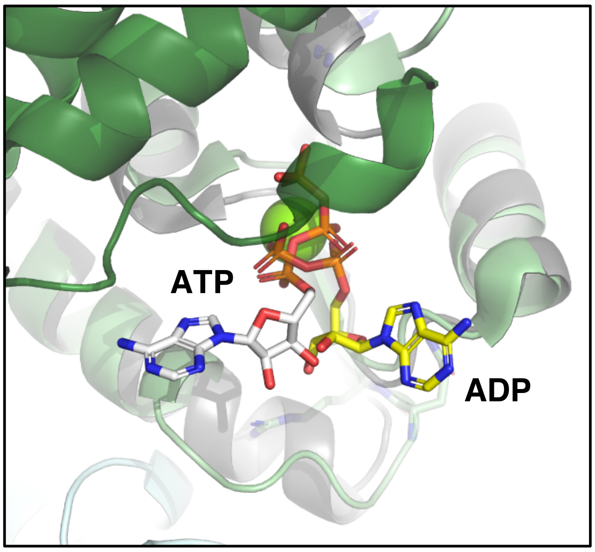

Supplementary Figure S1. Bacterial and mammalian PFKs have effector binding sites in equivalent positions, but they bind their effectors in different orientations. The high-affinity R state of *Geobacillus stearothermophilus* PFK is shown in pale grey with the allosteric activator, ADP (yellow), in the effector binding site (PDB ID: 4PFK). Superimposed is the low-affinity T state of human liver PFK (PFKL), which is shown in green with the allosteric inhibitor, ATP (white), in effector site 3 (PDB ID: 8W2H).

Supplementary Tables

Table S1. Comparison of steady-state kinetic parameters for PP_i-dependent PFKs catalysing forward and reverse reactions.

PFK source organism	Forward: F6P + PP_i → FBP + P_i			Reverse: FBP + P_i → F6P + PP_i			Ref.
	k_{cat} (s^{-1})	K_M^{F6P} (mM)	k_{cat}/K_M (s^{-1} M^{-1})	k_{cat} (s^{-1})	K_M^{FBP} (mM)	k_{cat}/K_M (s^{-1} M^{-1})	
Giardia lamblia	83	0.25	3.3×10^5	83	0.072	1.2×10^6	[1]
Methylobacterium nodulans	16	0.65	2.5×10^4	13	0.043	3.1×10^5	[2]
Methylosinus trichosporium	24	0.24	1.0×10^5	24	0.074	3.4×10^5	[2]

Table S2. Summary of Phosphofructokinase Superfamily structures in the PDB.

Organism	PDB ID	Notes	Ref.
**Bacterial ATP-dependent PFKs**			
Geobacillus stearothermophilus	3PFK	Phosphates in the active and effector sites.	[3]
	4PFK	R state with F6P & ADP in the active site; ADP in the effector site.	[3]
	6PFK	T state with 2-phosphoglycolic acid in the effector site.	[4]
	1MTO	W179Y/Y164W mutant; F6P in the active site.	[5]
	3U39	Apoenzyme.	[6]
	4I36	D12A mutant, apoenzyme in T state conformation.	[7]
	4I7E	D12A mutant; T state with PEP in the effector site.	[7]
	4I4I	T156A mutant; T state with PEP in the effector site.	[7]
Escherichia coli	1PFK	R state with FBP & ADP in the active site; ADP in the effector site.	[8]
	2PFK	Apoenzyme.	[9]
Lactobacillus delbrueckii	1ZXX	R state with crystallographic sulfates.	[10]
Bacillus subtilis	4A3S	Apoenzyme.	[11]
Staphylococcus aureus	5XOE	Apoenzyme.	[12]
	5XZ6	ATP analogue in the active site.	[12]
	5XZ7	F6P & glycerol in the active site.	[12]
	5XZ8	ATP analogue & F6P in the active site.	[12]
	5XZ9	ATP in the active site.	[12]
	5XZA	ADP in the active site.	[12]
**Eukaryotic ATP-dependent PFKs**			
Komagataella pastoris	3OPY	T state: ATP in effector site N1.	[13]
Saccharomyces cerevisiae	3O8O	R state: F6P in the active site and fructose 2,6-bisphosphate in effector site F'.	[14]
Oryctolagus cuniculus (muscle)	3O8L	ATP in the active site; ATP in effector site N1; ADP in N2.	[14]
	3O8N	ADP in the active site and in effector sites N1 & N2.	[14]
Homo sapiens (muscle)	4OMT	Apoenzyme.	[15]
Homo sapiens (platelet)	4RH3	ATP analogue in the active site.	[16]
	4U1R	ATP in the active site, phosphates at positions equivalent to the bacterial effector sites (E and E' sites).	[16]
	4WL0	Phosphates at the E and E' sites.	[16]
	4XZ2	F6P & ADP in the active site; FBP in effector site F'; phosphates at the E and E' sites.	[16]
	4XYJ	ATP in the active site and phosphates at the E and E' sites.	[17]
	4XYK	ADP in the active site and phosphates at the E and E' sites.	[17]
	7TFF	D564N mutant.	[18]
Homo sapiens (liver)	7LW1	F6P & ADP in the active site; FBP in effector site F'; agonist NA-11 in effector site N2.	[19]
	8W2G	R state tetramer. ADP & F6P in the active site. ADP in effector sites 1 and 2; FBP in the F' effector site.	[20]
	8W2H	T state tetramer. ATP in the active site. ATP in effector sites 2 and 3; FBP in the F' effector site.	[20]
	8W2J	T state filament (two stacked tetramers).	[20]
Trypanosoma brucei	2HIG	T state apoenzyme.	[21]
	3F5M	R state holoenzyme with ATP in the active site.	[22]
	6SY7	AMP in the effector site.	[23]
	6QU3	Allosteric inhibitor CTCB-360 bound.	[24]
	6QU4	Allosteric inhibitor CTCB-405 bound.	[24]
	6QU5	Allosteric inhibitor CTCB-12 bound.	[24]
**Bacterial PP$_i$-dependent PFKs**			
Borrelia burgdorferi	1KZH	With crystallographic sulfates.	[25]
	2F48	FBP in the active site.	None
Nitrosospira multiformis	3HNO	Apo enzyme.	None
Marinobacter aquaeolei	3K2Q	Apo enzyme.	None
**Eukaryotic PP$_i$-dependent PFK**			
Trichomonas vaginalis	9DQM	AMP & phosphate in putative effector sites.	None

Table S3. Identities of structurally equivalent positions that have been linked with phosphate donor specificity in PFKs, compiled by examining experimentally-determined structures.

Enzyme source	Residue in the GGD(D/G) motif	Residue in the P(G/K)TIDXD motif	Residue equivalent to N181 in *Bbu*PFK
**ATP-dependent PFKs**			
G. stearothermophilus	G104	G124	G108
E. coli	G104	G124	G108
L. delbrueckii	G104	G124	G108
B. subtilis	G104	G124	G108
S. aureus	G104	G126	G108
H. sapiens (muscle)	G120	G163	G124
H. sapiens (platelet)	G129	G172	G133
H. sapiens (liver)	G120	G163	G124
O. cuniculus	G120	G163	G124
*K. pastoris**	G315 / G287	G358 / G330	G319 / G291
*S. cerevisiae**	G310 / G302	G353 / G345	G314 / G306
T. brucei	G200	K226	G204
**PP_i-dependent PFKs**			
B. burgdorferi	D177	K203	N181
N. multiformis	D115	K141	T119
M. aquaeolei	D115	K141	T119
T. vaginalis	D115	K139	S119

*Residues identities for both the α subunit and the β subunit are listed.

Supplementary References

1 Phillips, N.F. and Li, Z. (1995) Kinetic mechanism of pyrophosphate-dependent phosphofructokinase from *Giardia lamblia*. *Mol. Biochem. Parasitol.* **73**, 43-51

2 Rozova, O.N., Khmelenina, V.N. and Trotsenko, Y.A. (2012) Characterization of recombinant PP_i-dependent 6-phosphofructokinases from *Methylosinus trichosporium* OB3b and *Methylobacterium nodulans* ORS 2060. *Biochemistry (Mosc.)* **77**, 288-295

3 Evans, P.R., Farrants, G.W. and Hudson, P.J. (1981) Phosphofructokinase: structure and control. *Philos. Trans. R. Soc. Lond. B Biol. Sci.* **293**, 53-62

4 Schirmer, T. and Evans, P.R. (1990) Structural basis of the allosteric behaviour of phosphofructokinase. *Nature* **343**, 140-145

5 Riley-Lovingshimer, M.R., Ronning, D.R., Sacchettini, J.C. and Reinhart, G.D. (2002) Reversible ligand-induced dissociation of a tryptophan-shift mutant of phosphofructokinase from *Bacillus stearothermophilus*. *Biochemistry* **41**, 12967-12974

6 Mosser, R., Reddy, M.C.M., Bruning, J.B., Sacchettini, J.C. and Reinhart, G.D. (2012) Structure of the apo form of *Bacillus stearothermophilus* phosphofructokinase. *Biochemistry* **51**, 769–775

7 Mosser, R., Reddy, M.C., Bruning, J.B., Sacchettini, J.C. and Reinhart, G.D. (2013) Redefining the role of the quaternary shift in *Bacillus stearothermophilus* phosphofructokinase. *Biochemistry* **52**, 5421-5429

8 Shirakiharat, Y. and Evans, P.R. (1988) Crystal structure of the complex of phosphofructokinase from *Escherichia coli* with its reaction products. *J. Mol. Biol.* **204**, 973-994

9 Rypniewski, W.R. and Evans, P.R. (1989) Crystal structure of unliganded phosphofructokinase from *Escherichia coli*. *J. Mol. Biol.* **207**, 805-821

10 Paricharttanakul, N.M., Ye, S., Menefee, A.L., Javid-Majd, F., Sacchettini, J.C. and Reinhart, G.D. (2005) Kinetic and structural characterization of phosphofructokinase from *Lactobacillus bulgaricus*. *Biochemistry* **44**, 15280-15286

11 Newman, J.A., Hewitt, L., Rodrigues, C., Solovyova, A.S., Harwood, C.R. and Lewis, R.J. (2012) Dissection of the network of interactions that links RNA processing with glycolysis in the *Bacillus subtilis* degradosome. *J. Mol. Biol.* **416**, 121-136

12 Tian, T., Wang, C., Wu, M., Zhang, X. and Zang, J. (2018) Structural insights into the regulation of *Staphylococcus aureus* phosphofructokinase by tetramer-dimer conversion. *Biochemistry* **57**, 4252-4262

13 Sträter, N., Marek, S., Kuettner, E.B., Kloos, M., Keim, A., Brüser, A. et al. (2011) Molecular architecture and structural basis of allosteric regulation of eukaryotic phosphofructokinases. *FASEB J.* **25**, 89-98

14 Banaszak, K., Mechin, I., Obmolova, G., Oldham, M., Chang, S.H., Ruiz, T. et al. (2011) The crystal structures of eukaryotic phosphofructokinases from baker's yeast and rabbit skeletal muscle. *J. Mol. Biol.* **407**, 284–297

15 Kloos, M., Brüser, A., Kirchberger, J., Schöneberg, T. and Sträter, N. (2014) Crystallization and preliminary crystallographic analysis of human muscle phosphofructokinase, the main regulator of glycolysis. *Acta Crystallogr. F Struct. Biol. Commun.* **70**, 578-582

16 Kloos, M., Brüser, A., Kirchberger, J., Schöneberg, T. and Sträter, N. (2015) Crystal structure of human platelet phosphofructokinase-1 locked in an activated conformation. *Biochem. J.* **469**, 421-432

17 Webb, B.A., Forouhar, F., Szu, F.E., Seetharaman, J., Tong, L. and Barber, D.L. (2015) Structures of human phosphofructokinase-1 and atomic basis of cancer-associated mutations. *Nature* **523**, 111-114

18 Voronkova, M.A., Hansen, H.L., Cooper, M.P., Miller, J., Sukumar, N., Geldenhuys, W.J. et al. (2023) Cancer-associated somatic mutations in human phosphofructokinase-1 reveal a critical electrostatic interaction for allosteric regulation of enzyme activity. *Biochem. J.* **480**, 1411-1427

19 Amara, N., Cooper, M.P., Voronkova, M.A., Webb, B.A., Lynch, E.M., Kollman, J.M. et al. (2021) Selective activation of PFKL suppresses the phagocytic oxidative burst. *Cell* **184**, 4480-4494

20 Lynch, E.M., Hansen, H., Salay, L., Cooper, M., Timr, S., Kollman, J.M. et al. (2024) Structural basis for allosteric regulation of human phosphofructokinase-1. *Nat. Commun.* **15**, 7323

21 Martinez-Oyanedel, J., McNae, I.W., Nowicki, M.W., Keillor, J.W., Michels, P.A., Fothergill-Gilmore, L.A. et al. (2007) The first crystal structure of phosphofructokinase from a eukaryote: *Trypanosoma brucei*. *J. Mol. Biol.* **366**, 1185-1198

22 McNae, I.W., Martinez-Oyanedel, J., Keillor, J.W., Michels, P.A., Fothergill-Gilmore, L.A. and Walkinshaw, M.D. (2009) The crystal structure of ATP-bound phosphofructokinase from *Trypanosoma brucei* reveals conformational transitions different from those of other phosphofructokinases. *J. Mol. Biol.* **385**, 1519-1533

23 Fernandes, P.M., Kinkead, J., McNae, I.W., Vásquez-Valdivieso, M., Wear, M.A., Michels, P.A.M. et al. (2020) Kinetic and structural studies of *Trypanosoma* and *Leishmania* phosphofructokinases show evolutionary divergence and identify AMP as a switch regulating glycolysis *versus* gluconeogenesis. *FEBS J.* **287**, 2847-2861

24 McNae, I.W., Kinkead, J., Malik, D., Yen, L.H., Walker, M.K., Swain, C. et al. (2021) Fast acting allosteric phosphofructokinase inhibitors block trypanosome glycolysis and cure acute African trypanosomiasis in mice. *Nat. Commun.* **12**, 1052

25 Moore, S.A., Ronimus, R.S., Roberson, R.S. and Morgan, H.W. (2002) The structure of a pyrophosphate-dependent phosphofructokinase from the Lyme disease spirochete *Borrelia burgdorferi*. *Structure* **10**, 659-671
